# Prognostic value of chemotherapy in addition to concurrent chemoradiotherapy in T3-4N0-1 nasopharyngeal carcinoma: a propensity score matching study

**DOI:** 10.18632/oncotarget.20014

**Published:** 2017-08-07

**Authors:** Li-Rong Wu, Hong-Liang Yu, Ning Jiang, Xue-Song Jiang, Dan Zong, Jing Wen, Lei Huang, Peng Xie, Wei Chen, Ting-Ting Wang, Da-Yong Gu, Peng-Wei Yan, Li Yin, Xia He

**Affiliations:** ^1^ Department of Radiation Oncology, Nanjing Medical University Affiliated Cancer Hospital, Jiangsu Cancer Hospital and Jiangsu Institute of Cancer Research, Nanjing 210009, China

**Keywords:** nasopharyngeal carcinoma, concurrent chemoradiotherapy, induction chemotherapy, adjuvant chemotherapy, prognosis

## Abstract

**Purpose:**

The objective of this study is to evaluate the contribution of induction (IC) or adjuvant (AC) chemotherapy additional to concurrent chemoradiotherapy (CCRT) for patients with T3-4N0-1 nasopharyngeal carcinoma (NPC) in the era of intensity-modulate radiotherapy (IMRT).

**Method and Materials:**

We retrospectively reviewed the data on 685 patients with newly diagnosed T3-4N0-1 NPC. Propensity score matching (PSM) method was used to match patients. Survival outcomes between different groups were calculated by Kaplan-Meier method and compared using log-rank test. Cox proportional hazard model was adopted to establish independent prognostic factors.

**Results:**

In total, 236 pairs were selected from the primary cohort. Univariate analysis revealed 3-year overall survival (OS) (90.8% vs. 90.3%, *P* = 0.820), distant failure-free survival (DFFS) (87.3% vs. 89.4%, *P* = 0.896) and locoregional failure-free survival (LRFFS) (95.4% vs. 93.0%, *P* = 0.311) rates were comparable between CCRT plus IC/AC and CCRT alone groups. Multivariate analysis found that treatment group was not an independent prognostic factors for OS (HR, 0.964; 95% CI, 0.620-1.499; *P* = 0.869), DFFS (HR, 1.036; 95% CI, 0.626-1.714; *P* = 0.890) and LRFFS (HR, 0.670; 95% CI, 0.338-1.327; *P* = 0.250). Further subgroup analysis according to overall stage also obtained similar results.

**Conclusion:**

Patients with T3-4N0-1 NPC receiving CCRT could not benefit from additional induction or adjuvant chemotherapy in the era of IMRT.

## INTRODUCTION

Nasopharyngeal carcinoma (NPC) is a head and neck malignancy that is endemic in South East Asia and Southern China [1–3], but relatively rare in Europe and the United States [2]. Unlike other head and neck cancers, radical surgery is not a treatment option for NPC because of its anatomic location. Nevertheless, NPC is highly sensitive to radiotherapy, and this treatment modality has been deemed the only curative strategy for non-disseminated disease. Treatment outcomes of early stage disease are usually excellent; however, the control of advanced NPC is challenging, and the condition has a 5-year overall survival (OS) of 67–79% [4, 5]. Therefore, much attention has been paid to locoregionally advanced disease, which accounts for 60–70% of all cases [6].

Since the Intergroup 0099 study [7] firstly reported a survival benefit from the combined strategy of radiotherapy with chemotherapy, concurrent chemoradiotherapy (CCRT) with or without adjuvant chemotherapy (AC) has been established as the standard treatment for locoregionally advanced NPC [8–13]. For the last two decades, induction chemotherapy (IC) administered before radiotherapy has received much attention [14–20] because it improves patient compliance and enables the early eradication of micrometastases. A recent network meta-analysis of individual patient data revealed that IC or AC in addition to CCRT can improve distant control or OS [21] in patients with advanced NPC. However, distant control and OS differ significantly between patients with different N stages [22]. Therefore, there is a lack of consensus among clinicians on the necessity of chemotherapy additional to CCRT in patients with N0-1-category locoregionally advanced NPC because this proportion of patients presents a relatively low rate of distant failure. Notably, previous studies usually recruited all patients with stage III and IVA-B disease and did not characterize this issue. This urgently needs to be addressed because additional of chemotherapy means more toxicities and heavier economic burden. Hence, we conducted this retrospective study to compare CCRT plus IC and/or AC with CCRT alone using propensity score matching (PSM) method [23].

## RESULTS

### Baseline characteristics

Among the primary cohort of 685 patients, there were 510 (74.5%) male and 175 (25.5%) female patients, carrying a ratio of 2.9:1. The median age was 49 (18-76) years-old. In total, 325 (47.4%) patients received CCRT alone and 360 (52.6%) patients received CCRT plus IC and/or AC. Additionally, 343 (50.1%) and 342 (49.9%) patients had stage III and IV disease, respectively. After matching, 236 pairs were selected by PSM and the baseline characteristics were summarized in Table 1. Obviously, host and tumor factors were well balanced between these two groups (*P* > 0.05 for all rates). Moreover, no significant difference was observed between patients receiving CCRT with or without IC/AC with regard to concurrent chemotherapy regimens (*P* = 0.771). Among the selected 236 patients, 156 (66.1%) patients received IC and 80 (33.9%) patients received AC. For IC, 58 (37.2%) and 98 (62.8%) patients received 2 and 3 cycles, respectively. For AC, most of patients received 2 cycles and only 20 (8.5%) patients received 3 cycles. With regard to concurrent chemotherapy, all the patients completed the assigned two cycles although some patients experienced dose reduction.

**Table 1:** Baseline characteristics of the 236 pairs with T3-4N0-1 NPC

Characteristics	CCRT plus IC/AC No. (%)	CCRT alone No. (%)	*P*
Median age (y, range)	48 (18-74)	50 (18-76)	0.156 ^a^
Gender			0.655 ^b^
Male	183 (77.5)	187 (79.2)	
Female	53 (22.5)	49 (20.8)	
Smoking			0.639 ^b^
Yes	97 (41.1)	92 (40.0)	
No	139 (58.9)	144 (60.0)	
Drinking			0.414 ^b^
Yes	34 (14.4)	28 (11.9)	
No	202 (85.6)	208 (88.1)	
KPS			0.918 ^b^
≥ 90	172 (72.9)	171 (72.5)	
≤ 80	64 (27.1)	65 (27.5)	
Median LDH (range, U/L)	177 (111-516)	171 (109-514)	0.893 ^a^
T category ^c^			0.519 ^b^
T3	123 (52.1)	116 (49.2)	
T4	113 (47.9)	120 (50.8)	
N category ^c^			0.896 ^b^
N0	34 (14.4)	35 (14.8)	
N1	202 (85.6)	201 (85.2)	
Overall stage ^c^			0519 ^b^
III	123 (52.1)	116 (49.2)	
IV	113 (47.9)	120 (50.8)	
Concurrent chemotherapy regimen			0.771 ^b^
PF	80 (33.9)	83 (35.2)	
TP	156 (66.1)	153 (64.8)	
Locoregional recurrence	15 (6.4)	20 (8.5)	0.380
Distant metastasis	32 (13.6)	31 (13.1)	0.892
Death	40 (16.9)	40 (16.9)	1.000

NPC = nasopharyngeal carcinoma; CCRT = concurrent chemoradiotherapy; IC = induction chemotherapy; AC = adjuvant chemotherapy; KPS = karnofsky performance score; LDH = lactate dehydrogenase; PF = cisplatin plus fluorouracil; TP = docetaxel plus cisplatin.

^a^ *P*-values were calculated by non-parametric test.

^b^ *P*-values were calculated by Chi-square test.

^c^ According to the 7^th^ edition of AJCC/UICC staging system.

### Treatment failure

By the last visit (July 20, 2016), 40 (8.5%) patients were lost to follow-up and the median follow-up duration for the selected 236 pairs was 51.1 months (range, 1.07-148.0 months). Overall, 20 (8.5%) in the CCRT alone group and 15 (6.4%) in the CCRT plus IC/AC group experienced locoregional recurrence (*P* = 0.380), and 31 (13.1%) in the CCRT group and 32 (13.6%) in the CCRT plus IC/AC group developed distant metastasis (*P* = 0.892). Consequently, 80 deaths in total were observed with 40 (16.9%) in each group (*P* = 1.000).

### Survival outcomes

**Figure 1:**
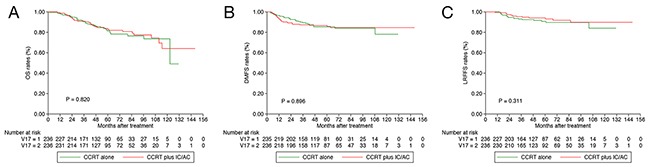
Kaplan-Meier OS **(A)**, DFFS **(B)** and LRFFS **(C)** curves for 236 pairs with T3-4N0-1 NPC stratifies as CCRT plus IC/AC and CCRT alone groups. OS = overall survival; DFFS = distant failure-free survival; LRFFS = locoregional failure-free survival; NPC = nasopharyngeal carcinoma; CCRT = concurrent chemoradiotherapy; IC = induction chemotherapy; AC = adjuvant chemotherapy.

The estimated 3-year OS, distant failure-free survival (DFFS) and locoregional failure-free survival (LRFFS) rates for the whole cohort were 90.6%, 88.4% and 94.2%, respectively. In comparison with the CCRT alone group, the CCRT plus IC/AC group achieved similar 3-year OS (90.8% vs. 90.3%, *P* = 0.820; Figure 1A), DFFS (87.3% vs. 89.4%, *P* = 0.896; Figure 1B) and LRFFS (95.4% vs. 93.0%, *P* = 0.311; Figure 1C) rates.

Multivariate analysis was performed to adjust for various factors and identify independent prognostic factors (Table 2). When entered into this model, treatment group (CCRT plus IC/AC vs. CCRT) was still not established as an independent prognostic factor for OS (HR, 0.964; 95% CI, 0.620-1.499; *P* = 0.869), DFFS (HR, 1.036; 95% CI, 0.626-1.714; *P* = 0.890) and LRFFS (HR, 0.670; 95% CI, 0.338-1.327; *P* = 0.250). Results of multivariate analysis including all the prognostic factors were summarized in Supplementary Table 1.

**Table 2:** Results of multivariate analysis for 236 pairs with T3-4N0-1 NPC

Endpoints	Variable	HR (95%CI)	*P*^a^
OS ^b^	Treatment group, CCRT plus IC/AC vs. CCRT alone	0.964 (0.620-1.499)	0.869
	Overall stage, IV vs. III	2.682 (1.289-5.580)	0.008
DFFS ^c^	Treatment group, CCRT plus IC/AC vs. CCRT alone	1.036 (0.626-1.714)	0.890
	Overall stage, IV vs. III	2.099 (1.139-3.868)	0.005
LRFFS ^d^	Treatment group, CCRT plus IC/AC vs. CCRT alone	0.670 (0.338-1.327)	0.250

NPC = nasopharyngeal carcinoma; OS = overall survival; DFFS = distant failure-free survival; LRFFS = locoregional failure-free survival; HR = hazard ratio; CI = confidence interval; CCRT = concurrent chemoradiotherapy; IC = induction chemotherapy; AC = adjuvant chemotherapy.

^a^ Multivariate *P*-values were calculated using an adjusted Cox proportional-hazards model with backward elimination and the following parameters: age (≥ 49y vs. < 49y), gender (male vs. female), smoking (yes vs. no), drinking (yes vs. no), KPS (≥ 90 vs. ≤ 80), N category (N1 vs. N0), overall stage (IV vs. III), concurrent chemotherapy regimen (TP vs. PF) and treatment group (CCRT plus IC/AC vs. CCRT).

^b^ Forty patients in the CCRT and 40 patients in the CCRT plus IC/AC were dead (*P* = 1.000).

^c^ Thirty-one in the CCRT and 32 patients in the CCRT plus IC/AC experienced distant metastasis (*P* = 0.892).

^d^ Twenty in the CCRT and 15 patients in the CCRT plus IC/AC developed locoregional recurrence (*P* = 0.380).

### Subgroup analysis

**Figure 2:**
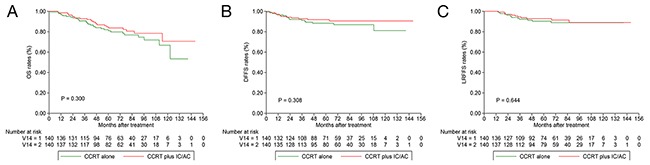
Kaplan-Meier OS **(A)**, DFFS **(B)** and LRFFS **(C)** curves for 140 pairs with T3N0-1 NPC stratifies as CCRT plus IC/AC and CCRT alone groups. OS = overall survival; DFFS = distant failure-free survival; LRFFS = locoregional failure-free survival; NPC = nasopharyngeal carcinoma; CCRT = concurrent chemoradiotherapy; IC = induction chemotherapy; AC = adjuvant chemotherapy.

As presented by the results of multivariate analysis, overall stage (IV vs. III) was a predictor for OS and DFFS. We therefore performed stratified analysis in patients with T3N0-1 and T4N0-1 disease; 140 and 152 pairs were selected by PSM. Among patients with T3N0-1 disease, the 3-year OS (92.8% vs. 90.6%; *P* = 0.300; Figure 2A), DFFS (92.7% vs. 91.1%; *P* = 0.308; Figure 2B) and LRFFS (94.6% vs. 92.2%; *P* = 0.644; Figure 2C) rates were comparable between CCRT plus IC/AC and CCRT alone groups. Multivariate analysis did not identify treatment group as an independent prognostic factor for all the endpoints (Table 3).

**Table 3:** Subgroup multivariate analysis stratified by overall stage

Endpoints	Variable	HR (95%CI)	*P*^a^
Stage III			
OS ^b^	Treatment group, CCRT plus IC/AC vs. CCRT alone	0.835 (0.488-1.430)	0.511
	Age, ≥ 49y vs. < 49y	2.074 (1.183-3.637)	0.011
DFFS ^c^	Treatment group, CCRT plus IC/AC vs. CCRT alone	0.712 (0.338-1.499)	0.372
LRFFS ^d^	Treatment group, CCRT plus IC/AC vs. CCRT alone	0.821 (0.367-1.835)	0.630
Stage IV			
OS ^b^	Treatment group, CCRT plus IC/AC vs. CCRT alone	1.234 (0.795-1.929)	0.344
	Age, ≥ 49y vs. < 49y	1.861 (1.157-2.992)	0.010
DFFS ^c^	Treatment group, CCRT plus IC/AC vs. CCRT alone	0.761 (0.452-1.284)	0.307
LRFFS ^d^	Treatment group, CCRT plus IC/AC vs. CCRT alone	0.822 (0.388-1.743)	0.609

OS = overall survival; DFFS = distant failure-free survival; LRFFS = locoregional failure-free survival; HR = hazard ratio; CI = confidence interval; CCRT = concurrent chemoradiotherapy; IC = induction chemotherapy; AC = adjuvant chemotherapy.

^a^ Multivariate *P*-values were calculated using an adjusted Cox proportional-hazards model with backward elimination and the following parameters: age (≥ 49y vs. < 49y), gender (male vs. female), smoking (yes vs. no), drinking (yes vs. no), KPS (≥ 90 vs. ≤ 80), N category (N1 vs. N0), concurrent chemotherapy regimen (TP vs. PF) and treatment group (CCRT plus IC/AC vs. CCRT).

^b^ Thirty-one in the CCRT and 24 patients in CCRT plus IC/AC with stage III (P = 0.292), and 38 in CCRT and 41 in CCRT plus IC/AC with stage IV (P = 0.695) were dead.

^c^ Seventeen in the CCRT and 12 patients in CCRT plus IC/AC with stage III (P = 0.327), and 33 in CCRT and 25 in CCRT plus IC/AC with stage IV (P = 0.243) experienced distant metastasis.

^d^ Thirteen in the CCRT and 11 patients in CCRT plus IC/AC with stage III (P = 0.669), and 16 in CCRT and 12 in CCRT plus IC/AC with stage IV (P = 0.428) developed locoregional recurrence.

**Figure 3:**
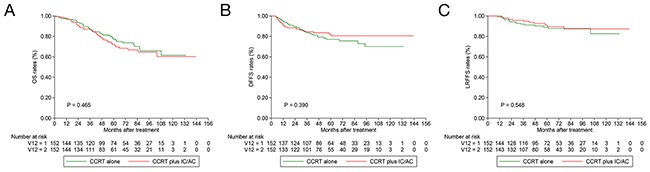
Kaplan-Meier OS **(A)**, DFFS **(B)** and LRFFS **(C)** curves for 152 pairs with T4N0-1 NPC stratifies as CCRT plus IC/AC and CCRT alone groups. OS = overall survival; DFFS = distant failure-free survival; LRFFS = locoregional failure-free survival; NPC = nasopharyngeal carcinoma; CCRT = concurrent chemoradiotherapy; IC = induction chemotherapy; AC = adjuvant chemotherapy.

With regard to patients with stage T4N0-1 disease, the 3-year OS (87.1% vs. 86.8%; *P* = 0.465; Figure 3A), DFFS (84.5% vs. 83.6%; *P* = 0.390; Figure 3B) and LRFFS (95.7% vs. 92.0%; *P* = 0.548; Figure 3C) rates did not differ significantly between CCRT plus IC/AC and CCRT alone groups. Similar as the results of univariate analysis, multivariate analysis revealed treatment group still had no prognostic value (Table 3).

## DISCUSSION

In our study, we firstly compared CCRT plus IC/AC with CCRT alone to evaluate the contribution of additional chemotherapy to CCRT in patients with T3-4N0-1 NPC, and the results revealed induction or adjuvant docetaxel plus cisplatin with fluorouracil (TPF) or docetaxel plus cisplatin (TP) could not further improve survival outcomes of 3-year OS, DFFS and LRFFS. Furthermore, stratified analysis according to overall stage also obtained similar results. By using PSM, potential bias may be avoided and the results should be reliable.

Currently, CCRT followed by AC is still the recommended treatment regimen for advanced NPC. Nevertheless, induction chemotherapy is also widely used at practice for its better compliance and early eradication of micrometastasis. Therefore, CCRT with IC or AC has come as a preferable treatment option at practice. However, distant control and overall survival significantly differ between patients with different N categories [22]. Therefore, it may be unlikely for all the advanced patients to receive the same treatment plan since they are at different risks. Possibly, patients with N0-1 category have lower tumor burden and therefore have better prognosis. Hence, additional chemotherapy to CCRT may be futile for this low-risk group. In our study, we clarified that patients with T3-4N0-1 disease could not benefit from IC or AC. In the study by International Nasopharynx Cancer Study Group [17], a positive effect of progression-free survival was achieved in patients with stage IV (≥ N2), indicating that high-risk patients would benefit from additional induction chemotherapy. Moreover, in the study by Chen et al. [11], no significantly survival difference was found between the CCRT plus AC and CCRT alone groups. This was mainly attributed to the high proportion (65%) of low-risk patients (stage III) recruited for the study, which diluted the survival benefit from AC. Given all these, patients with T3-4N0-1 disease should be grouped as low-risk, and additional chemotherapy as IC or AC should not be delivered.

Notably, although different regimens like TPF and TP were used as induction and adjuvant chemotherapy, it may be less likely to affect the conclusions of this study because both TP [16] and TPF [19] have been proven to be effective regimens in advanced NPC. Moreover, there is no evidence showing the efficacy difference between these two regimens. In our study, we combined induction and adjuvant chemotherapy together in the CCRT plus IC/AC group because the survival difference between induction-concurrent and concurrent-adjuvant chemotherapy sequences has not been clarified by any randomized trials so far. Possibly, they were equally effective as pointing out by a recent network meta-analysis [21]. Therefore, it should be reasonable to combine IC and AC together as a whole group. Concurrent chemotherapy used in our study were cisplatin-based regimens including TP and cisplatin plus fluorouracil (PF), which was firstly used in the study by Lin et al. [13]. Although different regimens were used, they were well balanced between CCRT plus IC/AC and CCRT alone groups. Moreover, multivariate analysis did not identify it as an independent prognostic factor. Therefore, the concurrent chemotherapy regimens would also have no impact on the conclusion.

The main strengthen of our study is the adoption of PSM method and multivariate analysis to evaluate the contribution of additional chemotherapy such as induction or/and adjuvant chemotherapy for patients with T3-4N0-1 disease; this could address the potential disadvantages of retrospective study like divergent confounders, selection bias and treatment heterogeneity [23]. Based on the results of this study, we suggested that CCRT may be enough for patients with T3-4N0-1 disease and additional chemotherapy could not bring further benefit. However, limitations of this study should also be acknowledged. First, the data was retrospectively collected from a single center. Second, the sample may be insufficient because patients with T3-4N0-1 have satisfactory survival outcomes and larger sample, especially in the subgroup analysis, is warrant to find out the difference. Third, the chemotherapy regimens used during CCRT is cisplatin-based double agents which may decrease the compliance. Therefore, the conclusions should be understood discreetly. Furthermore, powerfully prognostic biomarker like pre-treatment plasma Epstein-Barr virus (EBV) DNA [24–27] not considered because most of patients were treated at an early time when test of plasma EBV DNA was not available. In the studies by Peng et al. [28] and Du et al. [29], plasma EBV DNA was found to be an effective risk stratification factor. Therefore, future management of patients with T3-4N0-1 NPC should also take plasma EBV DNA into consideration.

## CONCLUSIONS

In summary, our study firstly showed that patients with T3-4N0-1 NPC receiving CCRT may not benefit from additional induction or adjuvant chemotherapy. Future prospective studies consisting large sample and plasma EBV DNA are warrant to confirm the results of this study.

## MATERIALS AND METHODS

### Study patient selection

We retrospectively reviewed the data on 685 consecutive patients with newly diagnosed, non-disseminated NPC treated at Nanjing Medical University Affiliated Cancer Hospital of China between May 2004 and October 2014. Patients meeting the following criteria were included in this study: (1) age 18 years or older; (2) T3-4N0-1 disease; (3) receiving CCRT with or without IC/AC; (4) no malignant tumor history and non-anticancer treatment previously. Written informed consent was obtained from all the patients before treatment, and this study was approved by the Research Ethics Committee of Jiangsu Cancer Hospital.

### Pre-treatment staging workup

In our hospital, patients at initial diagnosis received staging workup including clinical examinations of head and neck regions, fibreoptic nasopharyngoscopy, magnetic resonance imaging (MRI) or contrast-enhanced computed tomography (CT) of the head and neck to evaluate the extent of the primary tumor and regional lymph nodes. Bone scintigraphy, chest radiography or contrast-CT, and ultrasonography of the abdominal region would also be performed to identify distant metastasis. 18F-fluorodeoxyglucose (18F-FDG) positron emission tomography (PET)-CT would also be performed if clinically indicated, and 131 (19.1%) patients received this test. All patients were restaged according to the 7^th^ edition of the International Union against Cancer/American Joint Committee on Cancer (UICC/AJCC) system [30].

### Radiotherapy and chemotherapy

All the patients received radical intensity-modulated radiotherapy (IMRT) using simultaneous integrated boost (SIB) in our center. A total prescribed doses of 66–75Gy/31-35 fractions to the planning target volume (PTV) of primary gross tumor volume (GTVnx), 65–75Gy/32–35 fractions to the PTV of metastatic lymph nodes (GTVnd), 56–60Gy/30 fractions to the PTV of high-risk clinical target volume (CTV1) and 50Gy/30 fractions to the PTV of low-risk clinical target volume (CTV2) were delivered with first 30 fractions to CTV1/CTV2 and then a boost to PTV of GTVnx and GTVnd for patients with locally or regionally residual tumor after prescribed dose. In total, 10 (4.2%) patients in the IC/AC + CCRT group and 13 (5.1%) in the CCRT alone group received boost radiation dose (P = 0.662).

Induction or adjuvant chemotherapy were performed for using platinum-based regimens including docetaxel (75mg/m^2^ d1) with cisplatin (80mg/m^2^ in total for d1-3) or triplet of docetaxel (60mg/m^2^ d1) and cisplatin (80mg/m^2^ in total for d1-3) plus 5-fluorouracil (1000mg/m^2^/d d1-d5) every three weeks for 2 to 3 cycles. Concurrent chemotherapy regimens mainly consisted of cisplatin (80mg/m^2^ in total for d1-3) plus fluorouracil (400mg/m^2^/d d1-d4) or docetaxel (75mg/m^2^ d1) plus cisplatin (80mg/m^2^ in total for d1-3) at 3-week interval for two cycles.

### Follow-up

Follow-up was measured from first day of treatment to last examination or death. Patients were followed by clinical physical examination, nasopharyngoscopy, CT scan or MRI of the head and neck region, ultrasonography of the abdomen and chest X-ray every 3 months during the first 2 years, then every 6 to 12 months thereafter (or until death) even with last normal findings. The primary endpoint is OS (time to death from any reason), and other endpoints included DFFS (time to distant metastasis) and LRFFS (time to local or regional recurrence or both).

### Statistical analysis

PSM was performed using the Nearest Neighbor method at a 1:1 ratio. Logit estimation was used and the following variables were included to match patients with the caliper of 0.1: age, gender, smoking, drinking, karnofsky performance score (KPS), lactate dehydrogenase (LDH), T category, N category, overall stage and concurrent chemotherapy regimen. Failure events in two groups were not considered when matching patients. Non-parametric and Chi-square test was used to compare continuous and categorical variables between the two groups. Kaplan-Meier method was adopted to calculated survival outcomes and difference was compared by log-rank test. Multivariate analysis using the Cox proportional hazards model was performed to estimate hazard ratios (HRs), 95% confidence intervals (CIs) and identify independent prognostic factors.

## SUPPLEMENTARY MATERIALS TABLE


