# Supplementary material for: Prognostic value of chemotherapy in addition to concurrent chemoradiotherapy in T3-4N0-1 nasopharyngeal carcinoma: a propensity score matching study
Source: Oncotarget. 2017 Aug 7;8(44):76807–15. doi: 10.18632/oncotarget.20014 (PMC5652744; doi:10.18632/oncotarget.20014)
Supplement: Supplementary file 1 [file oncotarget-08-76807-s001.pdf]

Prognostic value of chemotherapy in addition to concurrent chemoradiotherapy in T3-4N0-1 nasopharyngeal carcinoma: a propensity score matching study

SUPPLEMENTARY MATERIALS

Supplementary Table 1: Results of multivariate analysis for 236 pairs with T3-4N0-1 NPC

Endpoints	Variable	HR (95%CI)	*P*[a]
OS [b]	Treatment group, CCRT plus IC/AC vs. CCRT alone	0.964 (0.620-1.499)	0.869
	Age, ≥ 49y vs. < 49y	1.021 (0.473-2.204)	0.957
	Gender, male vs. female	1.871 (0.892-3.922)	0.097
	Smoking, yes vs. no	0.713 (0.448-1.136)	0.155
	Drinking, yes vs. no	0.923 (0.414-2.055)	0.844
	KPS, ≥ 90 vs. ≤ 80	1.294 (0.778-2.153)	0.321
	N category, N1 vs. N0	1.260 (0.811-1.957)	0.304
	Overall stage, IV vs. III	2.682 (1.289-5.580)	0.008
DFFS [c]	Treatment group, CCRT plus IC/AC vs. CCRT alone	1.036 (0.626-1.714)	0.890
	Age, ≥ 49y vs. < 49y	0.737 (0.339-1.603)	0.442
	Gender, male vs. female	1.993 (0.949-4.189)	0.069
	Smoking, yes vs. no	0.781 (0.464-1.316)	0.354
	Drinking, yes vs. no	0.732 (0.285-1.880)	0.517
	KPS, ≥ 90 vs. ≤ 80	1.213 (0.691-2.131)	0.501
	N category, N1 vs. N0	1.365 (0.647-2.880)	0.413
	Overall stage, IV vs. III	2.099 (1.139-3.868)	0.005
LRFFS [d]	Treatment group, CCRT plus IC/AC vs. CCRT alone	0.670 (0.338-1.327)	0.250
	Age, ≥ 49y vs. < 49y	0.804 (0.411-1.573)	0.524
	Gender, male vs. female	2.301 (0.811-6.542)	0.117
	Smoking, yes vs. no	0.560 (0.197-1.590)	0.276
	Drinking, yes vs. no	0.868 (0.267-2.821)	0.814
	KPS, ≥ 90 vs. ≤ 80	0.712 (0.357-1.419)	0.335
	N category, N1 vs. N0	1.122 (0.428-2.943)	0.815
	Overall stage, IV vs. III	1.206 (0.614-2.373)	0.586

NPC = nasopharyngeal carcinoma; OS = overall survival; DFFS = distant failure-free survival; LRFFS = locoregional failure-free survival; HR = hazard ratio; CI = confidence interval; CCRT = concurrent chemoradiotherapy; IC = induction chemotherapy; AC = adjuvant chemotherapy.

[a] Multivariate *P*-values were calculated using an adjusted Cox proportional-hazards model with backward elimination and the following parameters: age (≥ 49y vs. < 49y), gender (male vs. female), smoking (yes vs. no), drinking (yes vs. no), KPS (≥ 90 vs. ≤ 80), N category (N1 vs. N0), overall stage (IV vs. III), concurrent chemotherapy regimen (TP vs. PF) and treatment group (CCRT plus IC/AC vs. CCRT).

[b] Forty patients in the CCRT and 40 patients in the CCRT plus IC/AC were dead ($P = 1.000$).

[c] Thirty-one in the CCRT and 32 patients in the CCRT plus IC/AC experienced distant metastasis ($P = 0.892$).

[d] Twenty in the CCRT and 15 patients in the CCRT plus IC/AC developed locoregional recurrence ($P = 0.380$).
